# *catena*-Poly[lead(II)-bis­(μ_2_-pyridazine-3-carboxyl­ato-κ^3^ *N* ^2^,*O*:*O*)]

**DOI:** 10.1107/S1600536810002199

**Published:** 2010-01-23

**Authors:** Wojciech Starosta, Janusz Leciejewicz

**Affiliations:** a Institute of Nuclear Chemistry and Technology, ul.Dorodna 16, 03-195 Warszawa, Poland

## Abstract

In the title structure, [Pb(C_5_H_3_N_2_O_2_)_2_]_*n*_, the Pb^II^ ion is six-coordinated by two pyridazine-3-carboxyl­ate ligands *via* N and O atoms, with the carboxyl­ato O atoms acting as bidentate and bridging adjacent Pb^II^ ions, giving rise to catenated mol­ecular ribbons propagating along the *a*-axis direction. The ribbons are connected by C—H⋯O hydrogen bonds and van der Waals inter­actions.

## Related literature

For the structures of 3*d*-metal and Mg(II) complexes with pyridazine-3-carboxyl­ate and water ligands containing monomeric mol­ecules with an octa­hedral enviroment for the metal ion, see: Ardiwinata *et al.* (1989 ▶), Gryz *et al.* (2003 ▶, 2004 ▶, 2006 ▶). Centrosymmetric dimeric mol­ecules, each with a different bridging mode, have been reported in the structure of a calcium(II) complex (Starosta & Leciejewicz, 2007 ▶), a uranyl complex (Leciejewicz & Starosta, 2009 ▶) as well as in the structure of a lead(II) complex with pyridazine-4-carboxyl­ate ligands (Starosta & Leciejewicz, 2009 ▶). For the structure of pyridazine-3-carboxylic acid hydro­chloride, see: Gryz *et al.* (2003 ▶).
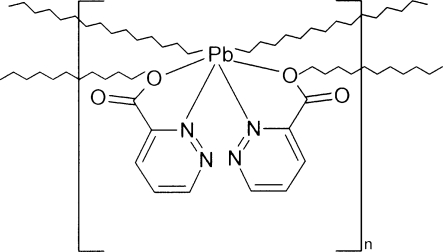


## Experimental

### 

#### Crystal data

[Pb(C_5_H_3_N_2_O_2_)_2_]
*M* *_r_* = 453.38
Monoclinic, 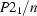

*a* = 8.0336 (16) Å
*b* = 10.386 (2) Å
*c* = 13.766 (3) Å
β = 93.72 (3)°
*V* = 1146.2 (4) Å^3^
*Z* = 4
Mo *K*α radiation
μ = 14.74 mm^−1^
*T* = 293 K
0.33 × 0.09 × 0.08 mm

#### Data collection

Kuma KM-4 four-circle diffractometer
Absorption correction: analytical (*CrysAlis RED*; Oxford Diffraction, 2008 ▶) *T*_min_ = 0.284, *T*_max_ = 0.379
3587 measured reflections
3365 independent reflections
2119 reflections with *I* > 2σ(*I*)
*R*_int_ = 0.040
3 standard reflections every 200 reflections  intensity decay: 1.3%

#### Refinement

*R*[*F* ^2^ > 2σ(*F* ^2^)] = 0.048
*wR*(*F* ^2^) = 0.137
*S* = 1.05
3365 reflections
172 parameters
H-atom parameters constrained
Δρ_max_ = 6.57 e Å^−3^
Δρ_min_ = −4.30 e Å^−3^

### 

Data collection: *KM-4 Software* (Kuma, 1996 ▶); cell refinement: *KM-4 Software*; data reduction: *DATAPROC* (Kuma, 2001 ▶); program(s) used to solve structure: *SHELXS97* (Sheldrick, 2008 ▶); program(s) used to refine structure: *SHELXL97* (Sheldrick, 2008 ▶); molecular graphics: *SHELXTL* (Sheldrick, 2008 ▶); software used to prepare material for publication: *SHELXL97*.

## Supplementary Material

Crystal structure: contains datablocks I, global. DOI: 10.1107/S1600536810002199/kp2247sup1.cif

Structure factors: contains datablocks I. DOI: 10.1107/S1600536810002199/kp2247Isup2.hkl

Additional supplementary materials:  crystallographic information; 3D view; checkCIF report

## Figures and Tables

**Table 1:** Selected bond lengths (Å)

Pb1—O21	2.492 (7)
Pb1—O11	2.569 (6)
Pb1—N12	2.645 (7)
Pb1—O21^i^	2.662 (7)
Pb1—O11^ii^	2.669 (6)
Pb1—N22	2.672 (6)

Symmetry codes: (i) 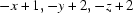; (ii) 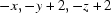.

**Table 2:** Hydrogen-bond geometry (Å, °)

*D*—H⋯*A*	*D*—H	H⋯*A*	*D*⋯*A*	*D*—H⋯*A*
C16—H16⋯O12^iii^	0.93	2.35	3.182 (12)	149
C14—H14⋯O21^iv^	0.93	2.76	3.489 (10)	136
C26—H26⋯O22^v^	0.93	2.42	3.201 (12)	142
C15—H15⋯O11^vi^	0.93	2.40	3.266 (10)	155
C25—H25⋯O12^vii^	0.93	2.42	3.328 (12)	165

Symmetry codes: (iii) 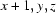; (iv) 
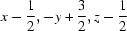
; (v) 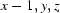; (vi) 
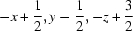
; (vii) 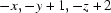.

**Fig. 1.**
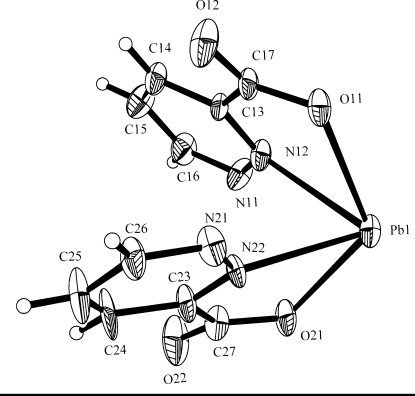
A structural unit of (1) with atom labelling scheme and 50% probability displacement ellipsoids.

**Fig. 2.**
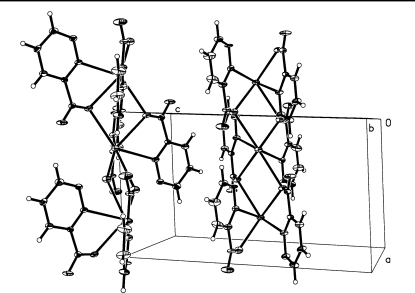
The alignement of two ribbons in the structure.

## supplementary crystallographic information

### Comment

In the structure of the title compound (I) each Pb^II^ ion is coordinated by two symmetry independent ligand molecules *via**N*,*O* atoms; their O atoms act as bidentate and bridging to adjacent metal ions (Fig. 1) to form molecular ribbons extending in the *a* direction (Fig.2). The second O atom of each carboxylato group does not participate in coordination. The coordination environment of a Pb^II^ ion involving O11,N12, O21, N22 and two bridging carboxylate O11^(I)^ and O21^(II)^ atoms (Table 1) is highly distorted. Both pyridazine rings are planar with r.m.s. of 0.0037 (2)Å and 0.0120((2)Å. The dihedral angle between the rings is 45.2 (1)°. Carboxylato planes make dihedral angles with the respective rings of 9.7 (1)° (C13/O11/O12) and of 8.8 (2)° (C23/O21/22). Bond distances and bond angles within both ligand molecules are in fair agreement with those reported for pyridazine-3-carboxylic acid chloride and other metal complexes with this ligand. The ribbons are held together by weak interactions between ring carbon atoms and carboxylato O atoms belonging to adjacent ribbons (Table 2).

### Experimental

2 mmols of pyridazine-3-carboxylic acid dissolved in 50 ml of hot water were boiled under reflux for three hours with small excess of lead hydroxide. After cooling to room temperature the mixture was filtered and left for crystallization. After evaporation to dryness, colourless single crystals were found on the bottom of the reaction vessel. They were separated, washed with cold ethanol and dried in air.

### Refinement

H atoms attached to pyridazine-ring C atoms were positioned geometrically and refined with a riding model using AFIX43 instruction. A maximum peak of 6.566 e Å^3^ and a deepest hole of -4.302 e Å^3^(each at 0.80 Å) were found on the final electron density map close to the Pb1 atom.

### Crystal data

**Table:**

[Pb(C_5_H_3_N_2_O_2_)_2_]	*F*(000) = 832
*M**_r_* = 453.38	*D*_x_ = 2.627 Mg m^−^^3^
Monoclinic, *P*2_1_/*n*	Mo *K*α radiation, λ = 0.71073 Å
*a* = 8.0336 (16) Å	Cell parameters from 25 reflections
*b* = 10.386 (2) Å	θ = 6–15°
*c* = 13.766 (3) Å	µ = 14.74 mm^−^^1^
β = 93.72 (3)°	*T* = 293 K
*V* = 1146.2 (4) Å^3^	Blocks, colourless
*Z* = 4	0.33 × 0.09 × 0.08 mm

### Data collection

**Table:**

Kuma KM-4 four-circle diffractometer	2119 reflections with *I* > 2σ(*I*)
Radiation source: fine-focus sealed tube	*R*_int_ = 0.040
graphite	θ_max_ = 30.1°, θ_min_ = 2.5°
profile data from ω/2θ scans	*h* = 0→11
Absorption correction: analytical (*CrysAlis RED*; Oxford Diffraction, 2008)	*k* = 0→14
*T*_min_ = 0.284, *T*_max_ = 0.379	*l* = −19→19
3587 measured reflections	3 standard reflections every 200 reflections
3365 independent reflections	intensity decay: 1.3%

### Refinement

**Table:**

Refinement on *F*^2^	Primary atom site location: structure-invariant direct methods
Least-squares matrix: full	Secondary atom site location: difference Fourier map
*R*[*F*^2^ > 2σ(*F*^2^)] = 0.048	Hydrogen site location: inferred from neighbouring sites
*wR*(*F*^2^) = 0.137	H-atom parameters constrained
*S* = 1.05	*w* = 1/[σ^2^(*F*_o_^2^) + (0.0903*P*)^2^] where *P* = (*F*_o_^2^ + 2*F*_c_^2^)/3
3365 reflections	(Δ/σ)_max_ = 0.002
172 parameters	Δρ_max_ = 6.57 e Å^−^^3^
0 restraints	Δρ_min_ = −4.30 e Å^−^^3^

### Special details

**Table:**

Geometry. All e.s.d.'s (except the e.s.d. in the dihedral angle between two l.s. planes) are estimated using the full covariance matrix. The cell e.s.d.'s are taken into account individually in the estimation of e.s.d.'s in distances, angles and torsion angles; correlations between e.s.d.'s in cell parameters are only used when they are defined by crystal symmetry. An approximate (isotropic) treatment of cell e.s.d.'s is used for estimating e.s.d.'s involving l.s. planes.
Refinement. Refinement of *F*^2^ against ALL reflections. The weighted *R*-factor *wR* and goodness of fit *S* are based on *F*^2^, conventional *R*-factors *R* are based on *F*, with *F* set to zero for negative *F*^2^. The threshold expression of *F*^2^ > σ(*F*^2^) is used only for calculating *R*-factors(gt) *etc*. and is not relevant to the choice of reflections for refinement. *R*-factors based on *F*^2^ are statistically about twice as large as those based on *F*, and *R*- factors based on ALL data will be even larger.

### Fractional atomic coordinates and isotropic or equivalent isotropic displacement parameters (Å^2^)

**Table:**

	*x*	*y*	*z*	*U*_iso_*/*U*_eq_	
Pb1	0.25237 (3)	1.03613 (3)	1.006155 (19)	0.02242 (12)	
O11	0.0180 (7)	0.9406 (6)	0.8924 (4)	0.0250 (12)	
N12	0.3296 (8)	0.8553 (6)	0.8814 (5)	0.0224 (13)	
N22	0.1664 (8)	0.8074 (6)	1.0764 (5)	0.0214 (13)	
O21	0.4781 (8)	0.9011 (6)	1.0862 (5)	0.0301 (13)	
N21	0.0078 (8)	0.7670 (7)	1.0745 (5)	0.0259 (15)	
O12	−0.0827 (8)	0.7546 (6)	0.8339 (6)	0.0388 (17)	
O22	0.5838 (9)	0.7035 (7)	1.1022 (7)	0.052 (2)	
N11	0.4876 (9)	0.8212 (8)	0.8732 (6)	0.0291 (16)	
C16	0.5237 (12)	0.7161 (9)	0.8257 (7)	0.033 (2)	
H16	0.6349	0.6937	0.8203	0.039*	
C14	0.2380 (10)	0.6694 (8)	0.7923 (6)	0.0257 (16)	
H14	0.1517	0.6183	0.7656	0.031*	
C26	−0.0251 (11)	0.6477 (9)	1.0961 (7)	0.0311 (19)	
H26	−0.1358	0.6214	1.0959	0.037*	
C17	0.0322 (10)	0.8273 (8)	0.8564 (6)	0.0207 (15)	
C13	0.2049 (9)	0.7810 (7)	0.8424 (5)	0.0182 (14)	
C23	0.2885 (10)	0.7262 (8)	1.0976 (6)	0.0260 (17)	
C15	0.3974 (11)	0.6369 (9)	0.7831 (6)	0.0316 (19)	
H15	0.4240	0.5631	0.7492	0.038*	
C27	0.4639 (10)	0.7783 (8)	1.0958 (6)	0.0256 (17)	
C24	0.2584 (14)	0.5976 (9)	1.1172 (9)	0.047 (3)	
H24	0.3460	0.5399	1.1286	0.056*	
C25	0.1003 (13)	0.5593 (10)	1.1192 (9)	0.045 (3)	
H25	0.0747	0.4751	1.1357	0.055*	

### Atomic displacement parameters (Å^2^)

**Table:**

	*U*^11^	*U*^22^	*U*^33^	*U*^12^	*U*^13^	*U*^23^
Pb1	0.01425 (17)	0.01657 (17)	0.03658 (19)	−0.00057 (14)	0.00282 (11)	−0.00095 (13)
O11	0.016 (3)	0.019 (3)	0.041 (3)	0.004 (2)	0.008 (2)	0.000 (2)
N12	0.013 (3)	0.018 (3)	0.037 (3)	0.001 (3)	0.004 (3)	−0.005 (3)
N22	0.018 (3)	0.014 (3)	0.032 (3)	0.000 (3)	0.005 (2)	0.007 (2)
O21	0.025 (3)	0.018 (3)	0.048 (3)	−0.008 (3)	0.004 (3)	0.004 (3)
N21	0.006 (3)	0.027 (4)	0.045 (4)	−0.005 (3)	0.003 (3)	0.008 (3)
O12	0.011 (3)	0.027 (3)	0.078 (5)	−0.006 (3)	0.004 (3)	−0.010 (3)
O22	0.024 (4)	0.030 (4)	0.101 (6)	0.012 (3)	0.002 (4)	0.001 (4)
N11	0.017 (4)	0.030 (4)	0.040 (4)	−0.004 (3)	0.003 (3)	−0.012 (3)
C16	0.022 (4)	0.028 (5)	0.048 (5)	0.012 (4)	−0.001 (4)	−0.007 (4)
C14	0.017 (4)	0.020 (4)	0.040 (4)	0.000 (3)	0.002 (3)	−0.007 (3)
C26	0.019 (4)	0.022 (4)	0.051 (5)	−0.009 (4)	0.000 (4)	0.008 (4)
C17	0.017 (4)	0.016 (4)	0.030 (4)	0.005 (3)	0.002 (3)	−0.002 (3)
C13	0.013 (3)	0.014 (3)	0.027 (3)	−0.002 (3)	0.000 (3)	−0.001 (3)
C23	0.017 (4)	0.017 (4)	0.045 (4)	−0.004 (3)	0.005 (3)	0.007 (3)
C15	0.022 (4)	0.033 (5)	0.040 (4)	0.004 (4)	0.009 (3)	−0.016 (4)
C27	0.010 (4)	0.024 (4)	0.044 (4)	0.001 (3)	0.004 (3)	−0.002 (3)
C24	0.031 (5)	0.014 (4)	0.093 (8)	0.005 (4)	−0.008 (5)	0.017 (5)
C25	0.028 (6)	0.017 (5)	0.092 (8)	0.001 (4)	0.010 (5)	0.012 (5)

### Geometric parameters (Å, °)

**Table:**

Pb1—O21	2.492 (7)	O22—C27	1.237 (10)
Pb1—O11	2.569 (6)	N11—C16	1.315 (12)
Pb1—N12	2.645 (7)	C16—C15	1.405 (12)
Pb1—O21^i^	2.662 (7)	C16—H16	0.9300
Pb1—O11^ii^	2.669 (6)	C14—C15	1.338 (12)
Pb1—N22	2.672 (6)	C14—C13	1.384 (11)
O11—C17	1.285 (10)	C14—H14	0.9300
O11—Pb1^ii^	2.669 (6)	C26—C25	1.385 (13)
N12—N11	1.330 (10)	C26—H26	0.9300
N12—C13	1.348 (9)	C17—C13	1.493 (11)
N22—C23	1.312 (10)	C23—C24	1.386 (12)
N22—N21	1.340 (9)	C23—C27	1.511 (12)
O21—C27	1.289 (10)	C15—H15	0.9300
O21—Pb1^i^	2.662 (7)	C24—C25	1.334 (14)
N21—C26	1.305 (11)	C24—H24	0.9300
O12—C17	1.217 (10)	C25—H25	0.9300
			
O21—Pb1—O11	122.4 (2)	N11—C16—H16	119.4
O21—Pb1—N12	72.1 (2)	C15—C16—H16	119.4
O11—Pb1—N12	61.55 (19)	C15—C14—C13	118.4 (8)
O21—Pb1—O21^i^	76.0 (2)	C15—C14—H14	120.8
O11—Pb1—O21^i^	112.9 (2)	C13—C14—H14	120.8
N12—Pb1—O21^i^	68.4 (2)	N21—C26—C25	121.8 (9)
O21—Pb1—O11^ii^	114.4 (2)	N21—C26—H26	119.1
O11—Pb1—O11^ii^	76.4 (2)	C25—C26—H26	119.1
N12—Pb1—O11^ii^	129.6 (2)	O12—C17—O11	125.6 (8)
O21^i^—Pb1—O11^ii^	160.5 (2)	O12—C17—C13	117.6 (7)
O21—Pb1—N22	62.5 (2)	O11—C17—C13	116.8 (7)
O11—Pb1—N22	71.4 (2)	N12—C13—C14	121.1 (7)
N12—Pb1—N22	71.4 (2)	N12—C13—C17	115.9 (6)
O21^i^—Pb1—N22	128.9 (2)	C14—C13—C17	123.0 (7)
O11^ii^—Pb1—N22	69.7 (2)	N22—C23—C24	121.7 (8)
C17—O11—Pb1	120.6 (5)	N22—C23—C27	116.8 (7)
C17—O11—Pb1^ii^	112.3 (5)	C24—C23—C27	121.5 (8)
Pb1—O11—Pb1^ii^	103.6 (2)	C14—C15—C16	118.9 (8)
N11—N12—C13	120.1 (7)	C14—C15—H15	120.6
N11—N12—Pb1	120.6 (5)	C16—C15—H15	120.6
C13—N12—Pb1	117.8 (5)	O22—C27—O21	123.7 (8)
C23—N22—N21	119.9 (7)	O22—C27—C23	119.8 (8)
C23—N22—Pb1	116.4 (5)	O21—C27—C23	116.5 (7)
N21—N22—Pb1	122.6 (5)	C25—C24—C23	118.0 (9)
C27—O21—Pb1	122.4 (5)	C25—C24—H24	121.0
C27—O21—Pb1^i^	111.8 (6)	C23—C24—H24	121.0
Pb1—O21—Pb1^i^	104.0 (2)	C24—C25—C26	118.5 (9)
C26—N21—N22	120.0 (8)	C24—C25—H25	120.8
C16—N11—N12	120.4 (7)	C26—C25—H25	120.8
N11—C16—C15	121.1 (8)		

Symmetry codes: (i) −*x*+1, −*y*+2, −*z*+2; (ii) −*x*, −*y*+2, −*z*+2.

### Hydrogen-bond geometry (Å, °)

**Table:**

*D*—H···*A*	*D*—H	H···*A*	*D*···*A*	*D*—H···*A*
C16—H16···O12^iii^	0.93	2.35	3.182 (12)	149
C14—H14···O21^iv^	0.93	2.76	3.489 (10)	136
C26—H26···O22^v^	0.93	2.42	3.201 (12)	142
C15—H15···O11^vi^	0.93	2.40	3.266 (10)	155
C25—H25···O12^vii^	0.93	2.42	3.328 (12)	165

Symmetry codes: (iii) *x*+1, *y*, *z*; (iv) *x*−1/2, −*y*+3/2, *z*−1/2; (v) *x*−1, *y*, *z*; (vi) −*x*+1/2, *y*−1/2, −*z*+3/2; (vii) −*x*, −*y*+1, −*z*+2.

## References

[bb1] Ardiwinata, E. S., Craig, D. C. & Philips, D. J. (1989). *Inorg. Chim. Acta*, **166**, 233–238.

[bb2] Gryz, M., Starosta, W. & Leciejewicz, J. (2004). *Acta Cryst.* E**60**, m1481–m1483.

[bb3] Gryz, M., Starosta, W. & Leciejewicz, J. (2006). *Acta Cryst.* E**62**, m123–m124.

[bb4] Gryz, M., Starosta, W., Ptasiewicz-Bąk, H. & Leciejewicz, J. (2003). *J. Coord. Chem.* **56**, 1505–1511.

[bb5] Kuma (1996). *KM-4 Software* Kuma Diffraction Ltd, Wrocław, Poland.

[bb6] Kuma (2001). *DATAPROC* Kuma Diffraction Ltd, Wrocław, Poland.

[bb7] Leciejewicz, J. & Starosta, W. (2009). *Acta Cryst.* E**65**, m94. 10.1107/S1600536808042219 PMC2967926 21581557

[bb8] Oxford Diffraction (2008). *CrysAlis RED* Oxford Diffraction Ltd, Yarnton, England.

[bb9] Sheldrick, G. M. (2008). *Acta Cryst.* A**64**, 112–122. 10.1107/S0108767307043930 18156677

[bb10] Starosta, W. & Leciejewicz, J. (2007). *Acta Cryst.* E**63**, m1662–m1663.

[bb11] Starosta, W. & Leciejewicz, J. (2009). *Acta Cryst.* E**65**, m1291. 10.1107/S1600536809039658 PMC2971413 21578059

